# Online Intervention Study - Willingness to donate organs among the employees of a German University

**DOI:** 10.1186/s40001-014-0043-y

**Published:** 2014-10-14

**Authors:** Matthias Heuer, Sonia Radunz, Friederike von Hugo, Carmen Kirchner, Natalie Wittenburg, Karl-Heinz Stammen, Andreas Paul, Gernot Kaiser

**Affiliations:** Department of General, Visceral- and Transplantation Surgery, University Hospital of Essen, Hufelandstr.55, 45122 Essen, Germany; The Center for Higher Education Development and Quality Enhancement, University of Duisburg-Essen, Duisburg, Germany

**Keywords:** Organ donation, Willingness, Knowledge, Survey, Online

## Abstract

**Background:**

Organ shortage remains a major challenge in transplantation medicine. The aim of this study was to analyze the public’s willingness to donate organs and to observe whether increased knowledge about organ donation has an effect on the attitude toward organ donation. The study in particular tested the efficacy of using electronic communication as a means to distribute information.

**Methods:**

In 2011, an Email invitation to participate in a survey was sent to the employees of the University Duisburg-Essen. The survey consisted of a two-piece questionnaire with an informational intervention on organ donation between the questionnaires. The technical design ensured that interviewees remained anonymous and could participate only once.

**Results:**

In total, 1,818 interviewees completed the questionnaire. Of the respondents, 42% were organ-donor card holders (which was consistent among genders and age groups), whereas 87% of the interviewees would support an organ donation for themselves. Of the interviewees who did not possess an organ-donor card, 67% were positively inclined toward holding one in future after reading the interventional information.

**Conclusions:**

The considerable improvement in attitude toward carrying an organ-donor card after reading the information illustrates the effectiveness of distributing concise information on organ donation. To increase the willingness to donate organs, it is of great importance to inform the public and facilitate the documentation of a decision to donate. The present study has proven the use of Email communication to be an important asset to this process.

## Background

“Organ donation saves lives”, a simple sentence that characterizes transplantation medicine. The worldwide lack of organ donors, however, creates an imbalance between the urgent need for transplants and the number of transplantations performed [1]. The answers to questions, such as how to make organ transplants available in adequate numbers and how to allocate scarce resources effectively and fairly, will determine the progress and success of transplantation medicine. The formation of a trusting attitude toward organ donation within a society increases the willingness to donate organs and thus is a key to future success in the field of transplantation [2,3].

German law provides that a declaration made by the deceased stating his or her willingness to donate or not to donate organs is legally decisive. If no declaration exists, this decision is left to the surviving ////dependents. Relatives who are unaware of the will of the deceased are twice as likely to decide against organ donation. This supports the notion that the formulation of one’s will regarding organ donation during one’s lifetime has a significant positive influence on transplantation rates.

So far, very few studies have examined the connection between education and the willingness to donate. In a pilot study in Great Britain, Exley *et al*. [4] showed, in a very small number of cases (*n* = 22), that lack of knowledge among the interviewees was the main cause for objecting to organ donation.

Surveys conducted at a German university hospital indicate that increased knowledge and education increase the willingness to donate organs. Radünz *et al*. [5] interviewed volunteers among the staff of the University Hospital Essen on the attitude toward organ donation. The results clearly showed that the high level of awareness in a hospital, including a transplantation center, increases the willingness of employees to hold organ-donor cards. Whereas at the time of the survey, a mere 17% of the overall population in Germany possessed an organ-donor card, 55% of the hospital staff interviewed (*n* = 242) had one. Of those in possession of a donor card, 19% were positively inclined toward carrying an organ-donor card in the future. In general, individuals in the medical field have a more-positive attitude toward organ donation [6].

The foremost prerequisites to increase the rate of organ donation are improved organizational structures that ensure transparency and a change of attitude through enhanced education [7]. The Organ Transplantation Act contains a provision to inform citizens about organ donation by using institutional means. It is the aim of such provision that citizens be informed continuously in such a sustained and institutionalized manner as is apt to promote and enhance citizens’ willingness to donate organs. It is important that the information provided is designed in such a way that each individual is called on to decide and manifest his or her decision regarding organ donation. A representative survey led by the Federal Centre of Health Education in 2010 showed that, although 74% of the interviewees would be willing to donate tissue or organs after death, only 25% actually possessed an organ-donor card in Germany [8].

But what is the best way to provide information to the public? Does electronically submitted information in combination with a questionnaire provide a reasonable method? Is a short information text useful? The present study analyzes the extent to which the information provided in the framework of an online intervention study is apt to change interviewees’ attitudes toward organ donation. Its aim is to investigate the possibility of using modern communication technologies to deliver purposeful, outcome-oriented information to promote organ donation. In addition, the intervention study provided several thousand people with essential information on the matter of organ donation.

## Methods

### Data resources and mode of questioning

In March 2011, an Email invitation to participate in an online survey was sent to all employees of the University Duisburg-Essen, of which 38% were regular personnel of the various departments (excluding the university hospital), 25% were supporting staff and lecturers, 15% were members of administrative, management, public relations, information and media services (ZIM), or library staff, and 22% were in the medical sector.

The participants in the survey were given 21 days to fill in the online questionnaire. An Email reminder was sent out 6 days before the deadline. The website allowed interviewees to remain anonymous and to participate only once. The online intervention study consisted of a two-piece questionnaire with an informational section between the questionnaires. The first part of the questionnaire and the informational section were the same for all interviewees. The second part of the questionnaire was based on answers from part one.

### Part One: questions on health

Part one of the survey consisted of 10 general questions regarding attitude toward and behavior regarding personal health. Subjects included diet, tobacco consumption, workout habits, medical checkups, influenza immunization, blood donation, execution of an advance medical directive, and ability to administer first aid. Additionally, participants were asked if they possessed an organ-donor card and whether they would be willing to become an organ donor.

### Part Two: intervention through information

Part two of the survey, the intervention, contained information on organ donation (498 words, one chart, and three screen pages). The information included the importance of organ donation, the donor situation in Germany compared with other countries, and the procedures involved in organ donation, including medical prerequisites (for example, brain death). The section explained the role of a donor card and touched on the emotional difficulties relatives face when attending the deathbed of a potential organ donor.

### Part three: adaptive questioning: reflection on interventional information

Part three asked the participants whether they had read the information, and then asked eight questions about the interventional information and its impact on the interviewee.

### Part four: personal data and link to information website

The final questions gathered personal data. A link to the homepage of the organ-donation task force of the University Hospital Essen (www.organspende-essen.de/) was provided, along with the option to print an organ-donor card. User frequency was documented.

### Statistics

The results were recorded in an Excel sheet and evaluated by using MS Excel tools, as well as the statistics software SPSS 15.0 (SPSS Inc., an IBM Company, Chicago, IL, USA). An evaluation of the hits on the homepage of the organ-donation task force at the University Hospital Essen was included.

Nominal data (for example, yes/no) were organized in contingency tables and analyzed by using the χ^2^ homogeneity test (n × m correlation to χ^2^ test for fourfold tables). Such a test examines whether the given data are distributed across the cells according to group strength (homogeneous) or not. Homogeneity was stated as the null hypothesis, and nonhomogeneity, as the alternative hypothesis. Probability of error was defined as ≤0.05.

## Results

In total, 1,818 interviewees filled in the complete questionnaire. Of the interviewees, 97.5% (*n* = 1,772) did so without interruption. The average time it took for respondents to answer all questions was less than 3 minutes (168 seconds): it took a mean of 38 seconds to fill in the first part of the questionnaire (10 questions on the subject matter of health), a mean of 76 seconds to read the informational intervention, a mean of 34 seconds to fill in the second part of the questionnaire (reflection of information), and a mean of 20 seconds to fill in personal data.

Of the total of 1,818 interviewees, 56.5% (*n* = 1,027) were aged between 18 and 35 years, 35% (*n* = 636) between 36 and 55 years, and around 8.4% (*n* = 152) were older than 55 years. Of the interviewees, 42% (*n* = 769) of the interviewees were men, and 58% (*n* = 1,045) were women.

### Evaluation of Part One of the questionnaire

Of the interviewees, 42% possessed an organ-donor card (Table 1). No significant variations were found between age groups or genders. A total of 87.2% supported an organ donation for themselves, with no significant variations between age groups or sexes.

**Table 1:** **Of the respondents, 42% (n =754) of the respondents having a donor card**

			**Gender** ^**a**^				**Age** ^**b**^				
			**None**	**Female**	**Male**	**Total**	**None**	**18 to – 35**	**36 to – 55**	**55 and older-**	**Total**
**Donor card**	None	*n*	2	0	0	2	2	0	0	0	2
		%	50	0	0	0.1	66.7	0	0	0	0.1
	Yes	*n*	0	322	432	754	0	436	262	56	754
		%	0	41.9	41.3	41.5	0	42.5	41.2	36.8	41.5
	No	*n*	2	447	613	1062	1	591	374	96	1,062
		%	50	58.1	58.7	58.4	33.3	57.5	58.8	63.2	58.4
	**Total**	*n*	4	769	1,045	1,818	3	1,027	636	152	1,818
		%	100				100				

Gender and age indicate no significant effect on holding a donor card.

χ^2^-test: ^a^ *P* = 0.820; ^b^ *P* = 0.414.

### Evaluation of Part Two of the questionnaire

In total, 85% of the interviewees read the interventional information. The percentage of female interviewees who read the information (87%) was significantly higher than that of the male interviewees (81%). Also, the age group of 55 years and older showed a significantly higher percentage (91%) of having read the information than the average (Table 2).

**Table 2:** **This question was set to the 930 respondents who had no donor card and had read the information text**

			**Gender** ^**a**^				**Age** ^**b**^				
			**None**	**Female**	**Male**	**Total**	**None**	**18 to 35**	**36to 55**	**55 and older**	**Total**
Based on the information - donor card	None	*n*	1	7	7	15	1	3	7	4	15
		%	50	1.9	1.3	1.6	50	0.6	2.1	4.7	1.6
	Yes	*n*	0	256	363	619	0	347	216	56	619
		%	0	67.9	65.9	66.6	0	68.4	64.5	65.1	66.6
	No	*n*	1	114	181	296	1	157	112	26	296
		%	50	30.2	32.8	31.8	50	31	33.4	30.2	31.8
	**Total**	*n*	2	377	551	930	2	507	335	86	930
		%	100				100				

Of them, 66% could imagine holding a donor card after reading the information. Gender and age indicate no significant effect on holding a donor card based on the interventional information.

χ^2^ test: ^a^ *P* = 0.435; ^b^ *P* = 0.661.

Of the interviewees who read the information, 88% stated that such information was sufficient for their needs; the percentage of female interviewees who found the information to be satisfactory was 4% higher than that of the male interviewees. About 15% of the interviewees stated that this was the first time ever they had reflected in more depth on the subject matter presented.

Of interviewees who initially stated that they objected to an organ donation for themselves, 20% (*n* = 196) supported organ donation after reading the information. In this group, the percentage of females (25%) was significantly higher than that of males (13%). Among the interviewees aged 55 years and older, only 16% changed their attitude after reading the information. Two thirds of the interviewees (66.6%) who did not yet possess an organ-donor card were positively inclined to hold one in the future. No significant differences were found with respect to age groups or sexes.

### Why are you still undecided with respect to organ donation?

This question was presented to 156 interviewees; 9% of the waverers needed more information; 37% named ethical or religious doubts as reasons for their indecisiveness; and another 37% stated that they were afraid. The percentage of female interviewees citing “fear” as a reason was notably higher than that of male interviewees. No significant variations were noted with respect to age groups.

Three fourths of the interviewees who objected to an organ donation for themselves did not object to the concept of organ donation in general.

Of the interviewees who possessed an organ donor card before participating in the survey, 84% confirmed that they felt reassured in their decision by the information provided. No difference was noted between gender groups, but positive responses were significantly higher among the younger age groups (*P* = 0.001).

### Correlation analysis

#### Adequate information: positive attitude toward donor card

Among those who deemed the information to be satisfactory, 70% were positively inclined toward holding an organ-donor card in future. Among those who responded that the information was inadequate, only 49% were positively inclined to hold an organ-donor card in future, the percentage being notably higher among the male interviewees (male interviewees, 56.3%, versus female interviewees, 45.1%) and also increasing with age (age group, 18 to 35: 41.6%; age group, 36 to 55: 56.6%; age group older than 55: 75.0%). The survey showed that adequate information is the most important criterion for a decision in favor of organ donation and the documentation of such decision.

### Impact of individual factors with respect to the possession of an organ-donor card/the inclination to become a donor-card carrier

#### Have you ever donated blood?

Of the blood donors, 48% also carry an organ-donor card. The survey shows that the willingness to carry an organ-donor card is significantly higher among blood donors (*P* < 0.001 and *P* = 0.027).

#### Do you smoke every day?

Of the interviewees, 279 stated that they smoke every day; 99 of these individuals possess an organ-donor card (35%). The survey shows that the number of organ-donor card holders is significantly lower among smokers (*P* = 0.026).

#### Do you go to recommended medical check-ups?

Of the interviewees who regularly go to medical check-ups, 43.5% carry an organ-donor card. The survey shows that the percentage of those who regularly go to medical check-ups is significantly higher among organ-donor card holders (*P* = 0.006).

#### Do you have an advance medical directive?

The results for this question show significant group variation. The percentage of interviewees who have executed an advance medical directive is significantly higher among organ-donor card carriers (*P* = 0.003) but also among those who show a negative inclination to carrying an organ-donor card in future (*P* < 0.001).

Of the 149 interviewees who have executed an advance medical directive, 53% also hold an organ-donor card. The interviewees possessing an advance medical directive were less influenced by the interventional information.

### Evaluation of the use of the home page of the organ-donation task force of the University Hospital Essen in the context of the survey

**Figure 1:**
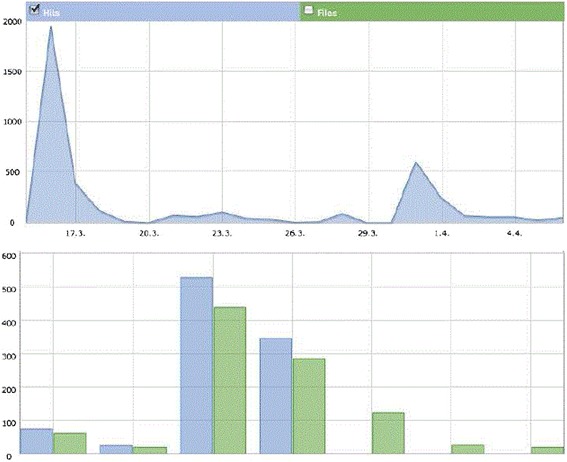
**Chart 1: The hits recorded on the home page (** www.organspende-essen.de/ **) between March 15, 2011, and April 6** ^**,**^ **2011.** The survey and a link to the home page were sent out on March 16, 2011, followed by an Email reminder on March 31, 2011.

The day the invitation to participate in the survey was sent out, the home page of the organ-donation task force of the University Hospital Essen recorded 1,950 hits (Figure 1). Thereafter, the number of hits decreased significantly (compare below chart). On normal days, about 60 hits are recorded on the home page. In March 2011, during the time of the survey, 111 organ-donor cards were downloaded from the home page, a large increase compared with the monthly average of fewer than 10 downloads.

## Discussion

The global shortage of organ donations poses a major challenge to transplantation medicine. It is society’s responsibility to find viable solutions to counteract the shortage of organ donors. Currently, 40% of potential organ donations in Germany are inhibited by relatives refusing to give consent, which illustrates the importance of educating families about organ donation before hospitalization or the death of their relatives. Awareness campaigns can create a positive image and increase the number of donations. A total of 1,818 interviewees completed the intervention study, thus awareness for these individuals was increased.

According to the present study, the factors of age and gender do not have a critical influence on the decision for or against organ donation. The comprehensive analysis of all data collected in the survey shows that the most decisive factor is one’s level of information. With respect to the groups surveyed, interventional information of less than 1.5 minutes sufficed in assuring 67% of the relevant interviewees to positively reconsider holding an organ-donor card. Considering that 42% of the interviewees already held an organ-donor card, the fact that two thirds of the remaining 58% could be positively motivated to consider holding one in future is a very encouraging finding. On conclusion of the survey, only a small percentage of interviewees remained unwilling to document a decision in favor of organ donation. Thus, the concise intervention provided a sufficient amount of knowledge to enhance the interviewees’ motivation to carry a donor card.

The increase of visits to the organ-donation task force of the University Hospital Essen home page (www.organspende-essen.de/) showed that the intervention motivated participants to seek further information. Additionally, the increase in organ-card downloads shows that the intervention encouraged participants to document their decision to become an organ donor. The study highlights the benefit of providing a simple strategy to deliver information and document one’s decision regarding organ donation, as people are responsive to such directives.

The use of modern communication media, such as Email, Twitter, and social networks, can be an expedient means of providing such opportunities. Online communication is cost-effective and allows rapid distribution of information to a large circle of recipients. Future studies should explore the Internet’s potential, in particular social networks, in increasing the willingness to donate organs and documenting the decision. A positive statement on organ donation posted on Twitter by teen star Justin Bieber, for example, resulted in more than 1,000 new registrations for organ donation on a United States Facebook website [9].

Of the interviewees who participated in the present survey, 42% were in possession of an organ-donor card, a percentage that is notably higher than the 25% rate indicated in current representative studies in Germany. One reason for the high percentage of donor-card carriers could be the above-average portion of people employed in the medical professions, as 22% of the interviewees worked in the healthcare sector. Another reason for the above-average percentage of organ-donor card holders could be the relatively high education level among university staff. This would reinforce the notion that greater knowledge relates to higher levels of organ donation. However, the high organ-donation acceptance may also be because the given positive answer can be influenced by the expectations of the society and was not always a reflection of the interviewee’s opinion during the survey.

The study found that a significantly high percentage of blood donors (48%) carry an organ-donor card, and a high percentage of blood donors (71%) not yet in possession of an organ-donor card are positively inclined to carrying one in the future. Therefore, blood drives should be considered for promoting organ donation in the future, as this population was found to be active and receptive regarding organ donation.

Another noteworthy finding was that only 8% of the interviewees have executed an advance medical directive, yet 53% of these individuals carry an organ-donor card. It seems reasonable to combine the execution of an advance medical directive with a documented decision on organ donation. The existence of an advance medical directive and organ donation provide legal certainty and relieve relatives of the burden of decision. These two initiatives should be associated with one another in future campaigns.

To increase the willingness to donate organs, it is of great importance to distribute relevant information and to facilitate the documentation of the decision [10,11]. Even a documentation of disapproval is a major help for relatives and clinical staff approaching the question of organ donation. We clearly point out that not all donor-card holders should be assumed to consent to organ donation, but a small subset also documents its disapproval.

## Conclusions

It is of great importance to inform the public and facilitate the documentation of a decision about organ donation. Therefore, the present study showed that the use of Email communication is an important asset to this process. Future studies should evaluate the success of the legally stipulated provision to provide institutionalized information and further to explore possible approaches to increase documentation of those willing to donate organs.
